# Prevalence of and risk factors for pulmonary complications after curative resection in otherwise healthy elderly patients with early stage lung cancer

**DOI:** 10.1186/s12931-019-1087-x

**Published:** 2019-07-04

**Authors:** Yunjoo Im, Hye Yun Park, Sumin Shin, Sun Hye Shin, Hyun Lee, Joong Hyun Ahn, Insuk Sohn, Jong Ho Cho, Hong Kwan Kim, Jae Ill Zo, Young Mog Shim, Ho Yun Lee, Jhingook Kim

**Affiliations:** 1 Division of Pulmonary and Critical Care Medicine, Department of Medicine, Samsung Medical Center, Sungkyunkwan University School of Medicine, Seoul, South Korea; 2 Department of Thoracic and Cardiovascular Surgery, Samsung Medical Center, Sungkyunkwan University School of Medicine, 81 Irwon-ro, Gangnam-gu, Seoul, 135-710 South Korea; 3 Division of Pulmonary Medicine and Allergy, Department of Internal Medicine, Hanyang University College of Medicine, Seoul, South Korea; 4 Statistics and Data Center, Samsung Medical Center, Seoul, South Korea; 5 Department of Radiology and Center for Imaging Science, Samsung Medical Center, Sungkyunkwan University School of Medicine, 81 Irwon-ro, Gangnam-gu, Seoul, 135-710 South Korea; 6 Department of Health Sciences and Technology, SAIHST, Sungkyunkwan University, Seoul, South Korea

**Keywords:** Elderly patients, Lung cancer, Postoperative pulmonary complications

## Abstract

**Background and objective:**

The prevalence of lung cancer has been increasing in healthy elderly patients with preserved pulmonary function and without underlying lung diseases. We aimed to determine the prevalence of and risk factors for postoperative pulmonary complications (PPCs) in healthy elderly patients with non-small cell lung cancer (NSCLC) to select optimal candidates for surgical resection in this subpopulation.

**Methods:**

We included 488 patients older than 70 years with normal spirometry results who underwent curative resection for NSCLC (stage IA-IIB) between 2012 and 2016.

**Results:**

The median (interquartile range) age of our cohort was 73 (71–76) years. Fifty-two patients (10.7%) had PPCs. Severe PPCs like acute respiratory distress syndrome, pneumonia, and respiratory failure had prevalences of 3.7, 3.7, and 1.4%, respectively. Compared to patients without PPCs, those with PPCs were more likely to be male and current smokers; have a lower body mass index (BMI), higher American Society of Anesthesiologists (ASA) classification, more interstitial lung abnormalities (ILAs), and higher emphysema index on computed tomography (CT); and have undergone pneumonectomy or bilobectomy (all *p <* 0.05). On multivariate analysis, ASA classification ≥3, lower BMI, ILA, and extent of resection were independently associated with PPC risk. The short-term all-cause mortality was significantly higher in patients with PPCs.

**Conclusions:**

Curative resection for NSCLC in healthy elderly patients appeared feasible with 10% PPCs. ASA classification ≥3, lower BMI, presence of ILA on CT, and larger extent of resection are predictors of PPC development, which guide treatment decision-making in these patients.

**Electronic supplementary material:**

The online version of this article (10.1186/s12931-019-1087-x) contains supplementary material, which is available to authorized users.

## Summary at a glance

Curative resection for NSCLC in healthy elderly patients appeared feasible with 10% PPC, which is comparable to NSCLC patients of all ages with preserved lung function. ASA classification, BMI, extent of surgery, and radiological abnormalities such as ILA were important determinants of PPCs in elderly patients with normal lung function.

## Introduction

The median age at diagnosis of lung cancer is 70 years, [1] and more than 70% of future lung cancer cases are expected to occur in adults older than 65 years [2]. As a result of early detection of lung cancer by screening [3], and the recent advances in surgical techniques and perioperative management, the need for curative surgical resection of early stage non-small cell lung cancer (NSCLC) in elderly patients has been increasing [4]. Given the current trend, surgeons are encountering larger numbers of elderly patients with surgically resectable NSCLC and have to assess the benefits and risks of surgical treatment in this population.

Postoperative pulmonary complications (PPCs) are the major cause of perioperative morbidity and mortality, occurring in 14-40% of patients after lung resection [5, 6]. Old age has been considered as a poor prognostic factor for PPCs due to the tendency for elderly patients to have multiple and inter-related risk factors, such as poor performance, underlying comorbidities, and impaired lung function [7, 8]. While decreased lung function is a major determinant for PPCs, and underlying lung diseases are associated with poorer surgical outcomes [9–13], healthy elderly patients with preserved lung function and no underlying lung diseases would be suitable candidates for surgery. In this population, however, the evidence to decide the treatment strategy is still scant, when comparing the risks between postsurgical mortality/morbidity and lung cancer progression [14]. Moreover, although chest computed tomography (CT) is performed in all patients with lung cancer, there is no consensus regarding preoperative chest CT evaluation to detect early structural abnormalities that are not reflected in pulmonary function tests. In this regard, it is imperative that we fully understand the risk factors for PPCs including chest CT findings particularly in healthy elderly patients with lung cancer.

Therefore, we aimed to assess the prevalence of PPCs after surgical resection of early stage NSCLC in healthy elderly patients. Furthermore, including the preoperative chest CT findings for thoroughly evaluating the pulmonary condition, we examined to identify independent risk factors for PPCs to select optimal candidates for curative lung resection in this population.

## Materials and methods

### Study population

The present study investigated surgical patients aged over 70 years, as this population is at high risk of PPCs following ACP guidelines [15, 16]. Thus, we conducted a retrospective cohort study of 511 patients older than 70 years with normal spirometry results who underwent curative resection for stage I and II NSCLC at Samsung Medical Center between January 2012 and December 2016. We excluded patients who previously underwent lung resection with lobectomy (*n* = 8), Ivor Lewis operation (*n* = 5), and those with interstitial lung disease (*n* = 10). In total, 488 patients were included in the final analysis. This study was approved by the Institutional Review Board of Samsung Medical Center, which exempted the requirement for informed consent as we only used de-identified data retrieved from electronic medical records (IRB no. 2018–06–032-001).

### Preoperative evaluation

Clinical characteristics, including age at surgery, smoking status, body mass index (BMI), comorbidity, tumor histology, pathologic stage, and type of surgery, were obtained from electronic medical records. Tumors were staged according to the 7th edition of the TNM classification [17].

The routine preoperative evaluation included spirometry and CT of the chest, positron emission tomography/CT, brain magnetic resonance imaging, and flexible bronchoscopy. Distant metastasis was also assessed by chest CT as radiographic study of the upper abdomen (i.e., liver, spleen, stomach and adrenal gland) was included in the chest CT examination for all patients. Spirometry and diffusing capacity of the lung for carbon monoxide (DLco) measurements were performed using Vmax 22 spirometers (SensorMedics, Yorba Linda, CA, USA) according to the American Thoracic Society/European Respiratory Society recommendations [18, 19]. Preserved pulmonary function was defined as a ratio of pre-bronchodilator forced expiratory volume in 1 s (FEV_1_) to forced vital capacity (FVC) > 0.70 and FVC ≥ 80% of the predicted value [20].

### CT findings

We analyzed and quantified the size and lobar location of the tumor, interstitial lung abnormalities (ILAs), emphysema index, superimposed infection, mosaic attenuation, airway abnormality, and ratio of main pulmonary artery-to-ascending aorta diameter on chest CT, which was performed closest to the operation, by using available software (Extended Brilliance Workspace v3.0; Philips Medical Systems, Cleveland, OH, USA).

ILAs were defined as particular patterns of increased lung density, including ground-glass or reticular abnormalities, diffuse centrilobular nodularity, nonemphysematous cysts, and honeycombing or traction bronchiectasis, affecting more than 5% of any lung zone [21]. ILAs were assessed subjectively by adding and scoring the overall extent of fibrosis-related lung parenchymal abnormalities, which were estimated from the nearest 5% of parenchymal involvement to the whole lung volume (100%) [22, 23]. We used an in-house computer software to assess and automatically calculate the emphysema index, which was defined as the volume fraction of the lung below − 950 HU [23, 24].

### Postoperative pulmonary complications

To prevent the occurrence of PPCs, every patient received postoperative respiratory physiotherapy program as well as early ambulation and lung expansion maneuvers, including deep breathing exercises, incentive spirometry, and airway clearance techniques such as active coughing and sputum expectoration. If the respiratory care was not effective, physiotherapist (specialized nurse for respiratory care) assessed the patient twice or three times per day, and actively conducted postural drainage and vibrator.

We considered the following PPCs that occurred during hospitalization or readmission for 60 days postoperatively: pneumonia, acute respiratory distress syndrome (ARDS), respiratory failure, significant atelectasis requiring bronchoscopy or reintubation, bronchopleural fistula/empyema, prolonged air leakage lasting for more than 5 days, and pneumothorax [25]. Severe PPCs were defined as ARDS, pneumonia, or respiratory failure. The definitions of PPCs are listed in Additional file 1: Table S1.

### Statistical analysis

Data are presented as n, median (interquartile range), or n (%). Categorical variables were compared using Pearson’s χ^2^ test, and continuous variables were compared using Student’s *t*-test. To identify risk factors independently associated with the occurrence of PPCs, we conducted a multivariate logistic regression, which included clinically relevant variables regardless of the *p*-value in the univariate analyses. Overall survival rate was estimated using the Kaplan–Meier method, and survival distributions between different groups were compared using the log-rank test.

All tests were two-sided, and a *p*-value of less than 0.05 was considered to indicate statistical significance. All statistical analyses were performed using IBM SPSS Statistics for Windows Version 24.0 (IBM Corp., Armonk, NY, USA).

## Results

### Patients and tumor characteristics

As shown in Tables 1 and 2, the median (interquartile range) age of patients was 73 (71–76) years and 47.7% were male. The median BMI of the patients was 24.1 kg/m^2^, and 43.4% of them were current or ex-smokers. Almost all (95.3%) patients had ASA classifications ≤ class 2. The common comorbidities included hypertension (52.7%), diabetes mellitus (21.5%), and previous (17.6%) or concurrent (8.6%) cancer. Among the CT findings, bronchiectasis (22.7%) and mosaic attenuation (19.5%) were common. Regarding tumor histology, adenocarcinoma was the most common (78.1%), followed by squamous cell carcinoma (16.6%). The majority of patients received lobectomy (75.8%) with systematic mediastinal lymph node dissection (86.1%) under video-assisted thoracic surgery (72.3%).

**Table 1:** Characteristics of study participants according to PPC development (*n* = 488)

	All (*n* = 488)	No PPCs (*n* = 436)	PPCs (*n* = 52)	*p*-value
Age, yr	73 (71–76)	73 (71–75)	73 (71–76)	0.427
Sex, male	233 (47.7)	198 (45.4)	35 (67.3)	**0.003**
BMI (kg/m^2^)	24.1 (22.1–25.9)	24.2 (22.2–26.1)	23.3 (20.7–25.1)	**0.007**
Smoking status				**0.013**
Current smoker	160 (32.8)	134 (30.8)	26 (50.0)	
Former smoker	51 (10.5)	45 (10.3)	6 (11.5)	
Never smoker	276 (56.6)	256 (58.9)	20 (38.5)	
ASA classification				**0.021**
1	32 (6.6)	31 (7.1)	1 (1.9)	
2	433 (88.7)	388 (89.0)	45 (86.5)	
≥ 3	23 (4.7)	17 (3.9)	6 (11.5)	
Comorbidity				
Diabetes mellitus	105 (21.5)	95 (21.8)	10 (19.2)	0.806
Hypertension	257 (52.7)	230 (52.8)	27 (51.9)	0.999
Congestive heart failure	3 (0.6)	3 (0.7)	0	0.999
Ischemic heart disease	37 (7.6)	33 (7.6)	4 (7.7)	0.999
Atrial fibrillation	21 (4.3)	19 (4.4)	2 (3.8)	0.999
Previous cancer history	86 (17.6)	72 (16.5)	14 (26.9)	0.095
Double primary cancer	42 (8.6)	37 (8.5)	5 (9.6)	0.990
Laboratory finding				
Serum hemoglobin (g/dL)	13.0 (12.3–14.0)	13.6 (12.6–14.5)	12.5 (11.6–14.3)	0.065
Serum albumin (g/dL)	4.3 (4.1–4.5)	4.3 (4.0–4.5)	4.2 (4.0–4.4)	0.614
Pulmonary function test				
FVC (% pred)	92 (85–100)	90 (84–98)	89 (83–96)	0.061
FEV_1_ (% pred)	101 (93–110)	100 (92–109)	99 (91–109)	0.579
FEV_1_/FVC	76 (73–79)	75 (72–77)	76 (72–78)	0.430
DL_CO_ (% pred) (*N* = 388)	111 (104–118)	111 (104–119)	108 (107–113)	0.869
CT finding				
Interstitial lung abnormality (%)	1.38 (4.14)	1.19 (3.57)	2.92 (7.22)	**0.004**
Emphysema index (%)	2.49 (7.61)	2.16 (6.97)	5.21 (11.4)	**0.006**
Superimposed infection	42 (8.6)	38 (8.7)	4 (7.7)	0.999
Mosaic attenuation	95 (19.5)	87 (20.0)	8 (15.4)	0.548
Bronchial wall thickening	15 (3.1)	14 (3.2)	1 (1.9)	0.933
Bronchiectasis	111 (22.7)	101 (23.2)	10 (19.2)	0.642
Diameter of MPA	28.0 (3.64)	28.0 (3.66)	27.8 (3.51)	0.637
Diameter of AA	39.0 (4.08)	39.1 (4.14)	38.1 (3.45)	0.092
Diameter of MPA to AA > 1	3 (0.6)	3 (0.7)	0	0.999

Data are presented as n, median (interquartile range), or n (%). *AA*: ascending aorta; *ASA*: American Society of Anesthesiologists; *BMI*: Body mass index; *CT*: Computed tomography; *FEV*_*1*_: Forced expiratory volume in 1 s; *FVC*: Forced vital capacity; *DL*_*CO*_: Diffusing capacity of the lung for carbon monoxide; *MPA*: Main pulmonary artery; *PPCs*: postoperative pulmonary complications; pred: predicted

**Table 2:** Characteristics of the tumor and surgical procedures in study participants according to PPC development (*n* = 488)

	All (*n* = 488)	No PPCs (*n* = 436)	PPCs (*n* = 52)	*p*-value
Tumor histology				**0.003**
Adenocarcinoma	381 (78.1)	350 (80.3)	31 (59.6)	
Squamous cell carcinoma	81 (16.6)	65 (14.9)	16 (30.8)	
Other NSCLCs	26 (5.3)	21 (4.8)	5 (9.6)	
Tumor location				0.186
Right upper lobe	164 (33.6)	147 (33.7)	17 (32.7)	
Right middle lobe	34 (7.0)	31 (7.1)	3 (5.8)	
Right lower lobe	99 (20.3)	82 (18.8)	17 (32.7)	
Left upper lobe	108 (22.1)	100 (22.9)	8 (15.4)	
Left lower lobe	83 (17.0)	76 (17.4)	7 (13.5)	
Tumor staging				
T stage				0.296
cT1a	120 (24.6)	109 (25.0)	11 (21.2)	
cT1b	136 (27.9)	125 (28.7)	11 (21.2)	
cT2a	163 (33.4)	145 (33.3)	18 (34.6)	
cT2b	39 (8.0)	33 (7.6)	6 (11.5)	
cT3	30 (6.1)	24 (5.5)	6 (11.5)	
N stage				0.833
cN0	458 (93.9)	409(93.8)	49 (94.2)	
cN1	30 (6.1)	26(6.2)	3 (5.8)	
Extent of surgery				**0.005**
Pneumonectomy & Bilobectomy	18 (3.7)	12 (2.8)	6 (11.5)	
Lobectomy	370 (75.8)	332 (76.1)	38 (73.1)	
Segmentectomy & Wedge resection	100 (20.5)	92 (21.1)	8 (15.4)	
VATS	353 (72.3)	321 (73.6)	32 (61.5)	0.093
MLND (%)				0.992
None	30 (6.1)	27 (6.2)	3 (5.8)	
Selective	38 (7.8)	34 (7.8)	4 (7.7)	
Systematic	420 (86.1)	375 (86.0)	45 (86.5)	

Data are presented as n (%). *MLND*: Mediastinal lymph node dissection; *NSCLC*: Non-small cell lung cancer; *PPCs*: Postoperative pulmonary complications; *VATS*: Video-assisted thoracoscopic surgery

### Development of PPCs

**Fig. 1:**
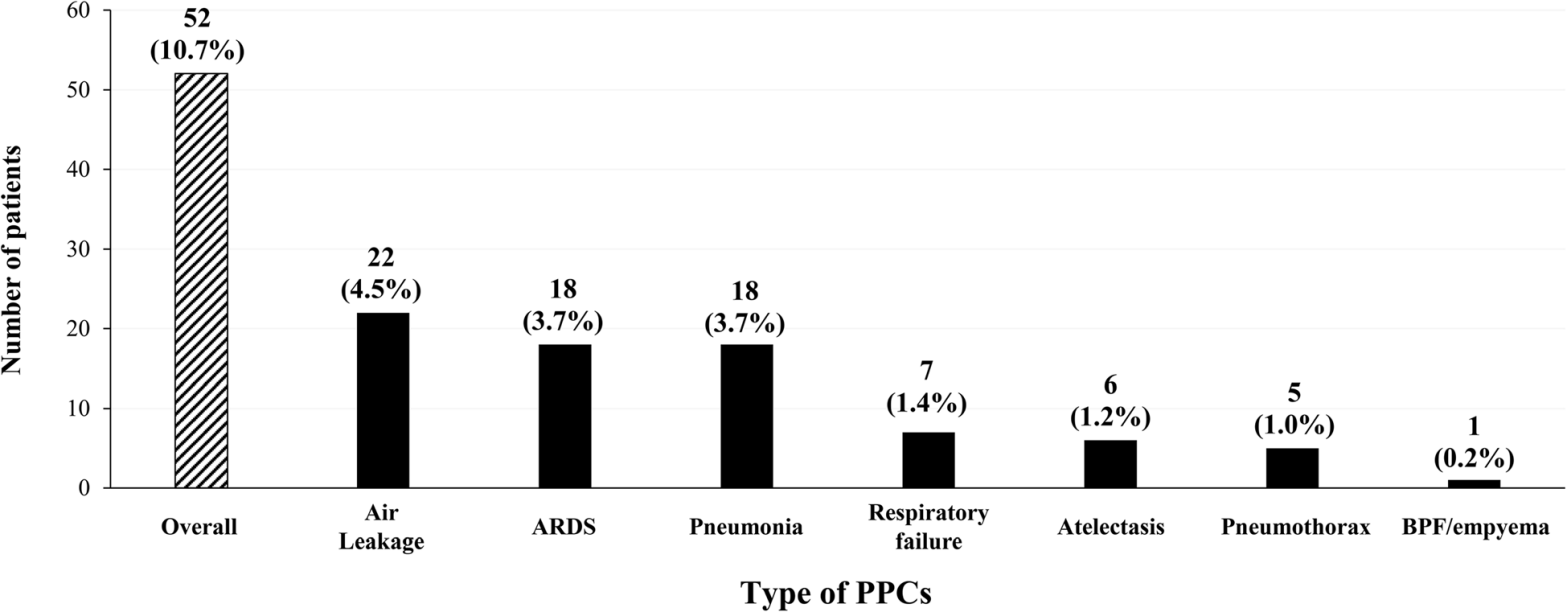
Incidence of each type of postoperative pulmonary complication. ARDS: acute respiratory distress syndrome; BPF: bronchopleural fistula

During the study period, 52 (10.7%) patients developed 77 PPCs following pulmonary resection. Among the 52 patients, the most common type of PPC was prolonged air leakage (*n* = 22, 42.3%), followed by ARDS (*n* = 18, 34.6%), pneumonia (n = 18, 34.6%), respiratory failure (*n* = 7, 13.5%), atelectasis (*n* = 6, 11.5%), and pneumothorax (*n* = 6, 11.5%) **(**Fig. 1**)**. Among the 52 patients with PPCs, 1 (1.9%), 2 (5.8%), and 1 (7.7%) died at 30, 90, and 180 days after surgery, respectively, and the 90-day and 180-day all-cause mortality rates were significantly higher in patients with PPCs than in those without PPCs (Table 3). The PPCs in these four patients were respiratory failure combined with ARDS, respiratory failure combined with pneumonia, aspiration pneumonia, and empyema.

**Table 3:** All-cause mortality at 30, 90, and 180 days according to PPCs

	30-day mortality	90-day mortality	180-day mortality
No PPC (*n* = 436)	0 (0/436)	0.23 (1/434)	0.69 (2/431)
PPC (*n* = 52)	1.9 (1/50)	5.77 (2/48)	7.7(1/47)
*p*-value	**0.031**	**0.016**	**0.008**

Data are presented as number (%)

*PPCs* = postoperative pulmonary complications

### Factors associated with PPC development

When the 52 patients with PPCs were compared to the 436 patients without PPCs, those with PPCs were more likely to be male (*p* = 0.003), current or ex-smokers (*p* = 0.013), have a lower BMI (*p* = 0.007), have an ASA classification ≥3 (*p* = 0.021), have more ILAs (*p* = 0.004), and have a higher emphysema index (*p* = 0.006). However, no significant differences were observed in age; comorbidities; serum hemoglobin and albumin levels; spirometry and DLco results; and other CT findings (Table 1). As shown in Table 2, while the proportion of patients with adenocarcinoma was higher among those without PPCs, the proportion of patients with squamous cell carcinoma was higher among those with PPCs (30.8% [16/52] vs. 14.9% [65/437]; *p* = 0.003). However, no significant difference was observed in tumor location or tumor stage between the two groups. Regarding surgical procedures, the patients with PPCs were more likely to have undergone pneumonectomy or bilobectomy than were those without PPCs (11.5% [6/52] vs. 2.8% [12/437]; *p* = 0.005). However, no significant difference was observed in the proportion of video-assisted thoracic surgery or extent of lymph node dissection between the two groups (Table 2).

A multivariate logistic regression model for evaluating risk factors for the occurrence of PPCs is shown in Table 4. Among the potential risk factors, ASA classification ≥3 (adjusted odds ratio [OR], 1.91; 95% confidence interval [CI], 1.14–3.21; *p* = 0.014), lower BMI (adjusted OR, 0.86; 95% CI, 0.78–0.95; *p* = 0.004), the presence of ILA (adjusted OR, 1.91; 95% CI, 1.02–1.13; *p* = 0.004), and pneumonectomy or bilobectomy (adjusted OR, 4.46; 95% CI, 1.54–12.9; *p*  =  0.006) were independently associated with PPC occurrence. When analysis was restricted to severe PPCs, which were defined as ARDS, pneumonia, or respiratory failure, lower BMI (adjusted OR, 0.85; 95% CI, 0.74–0.98; *p* = 0.025), the presence of ILA (adjusted OR, 1.09; 95% CI, 1.03–1.16; *p* = 0.002), and pneumonectomy or bilobectomy (adjusted OR, 5.57; 95% CI, 1.63–19.0; *p* = 0.006) were still associated with PPC occurrence (Table 5).

**Table 4:** Risk factors associated with PPCs

	Univariate analysis		Multivariate analysis	
Variable	OR (95% CI)	*p*-value	OR (95% CI)	*p*-value
Age	0.96 (0.88–1.06)	0.427		
Sex, male	2.47 (1.35–4.55)	**0.004**		
Smoking history (current & former)	2.25 (1.25–4.07)	**0.007**		
ASA classification ≥3	1.79 (1.10–2.93)	**0.019**	1.91 (1.14–3.21)	**0.014**
BMI	1.15 (1.05–1.29)	**0.003**	0.86 (0.78–0.95)	**0.004**
Hemoglobin	1.18 (0.95–1.46)	0.066		
Squamous vs. others	2.54 (1.33–4.84)	**0.005**		
ILA	1.07 (1.02–1.12)	**0.009**	1.91 (1.02–1.13)	**0.004**
Emphysema index	1.04 (1.01–1.07)	**0.010**		
Extent of surgery				
Segmentectomy & Wedge resection & Lobectomy	Reference		Reference	
Pneumonectomy & Bilobectomy	4.61 (1.65–12.8)	**0.004**	4.46 (1.54–12.9)	**0.006**

*ASA*: American Society of Anesthesiologists; *BMI*: body mass index; *CI*: confidence interval; *ILA*: interstitial lung abnormality; *OR*: odds ratio; *PPCs*: postoperative pulmonary complications

**Table 5:** Risk factors associated with severe PPCs (ARDS/pneumonia/respiratory failure) in this study

	Univariate analysis		Multivariate analysis	
Variable	OR (95% CI)	*p*-value	OR (95% CI)	*p*-value
Age	1.02 (0.91–1.14)	0.781		
Sex, male	2.29 (1.01–5.20)	**0.048**		
Smoking history (current & former)	1.95 (0.89–4.29)	0.098		
ASA classification ≥3	1.29 (0.61–2.75)	0.501		
BMI	0.85 (0.74–0.97)	**0.015**	0.86 (0.74–1.01)	**0.052**
Hemoglobin	0.76 (0.57–1.01)	**0.066**		
Squamous vs. others	1.85 (0.75–4.48)	0.186		
ILA	1.09 (1.03–1.15)	**0.003**	1.09 (1.03–1.16)	**0.003**
Emphysema index	1.03 (0.99–1.07)	0.067		
Extent of surgery				
Segmentectomy & Wedge resection & Lobectomy	Reference		Reference	
Pneumonectomy & Bilobectomy	5.55 (1.69–18.2)	**0.005**	5.57 (1.63–19.0)	**0.006**

*ARDS*: Acute respiratory distress syndrome; *ASA*: American Society of Anesthesiologists; *BMI*: Body mass index; *CI*: Confidence interval; *ILA*: Interstitial lung abnormality; *OR*: Odds ratio; *PPCs*: Postoperative pulmonary complications

There were 470 (96.3%) patients who underwent lobectomy, segmentectomy or wedge resection in our study population. Among them, lower BMI (adjusted OR, 0.86; 95% CI, 0.78–0.95; *p* = 0.005), and the presence of ILA (adjusted OR, 1.07; 95% CI, 1.02–1.13; *p* = 0.008) were independently associated with PPC occurrence, analyzed by multivariate logistic regression (Additional file 2: Table S2).

## Discussion

Our study showed that the overall prevalence of PPCs following lung resection was 10% for patients with early stage NSCLC aged over 70 years old with preserved lung function. Among the factors associated with PPC occurrence, we found several independent predictors of PPCs: ASA classification ≥3, lower BMI, ILAs on chest CT, and greater surgical extent.

PPC incidence after pulmonary resection ranged from 13 to 16% in prospective observational studies that included patients of all ages and with poor lung function [5, 26, 27]. Our study included only elderly patients with preserved lung function and showed a 10% overall incidence of PPCs after surgery for early stage NSCLC. This result is comparable to that of a previous study showing 10% PPCs after lung resection for NSCLC in patients of all ages with preserved lung function (median age, 64 years; range, 27–84 years) [27]. In addition, age over 70 years was not identified as a risk factor for PPCs in our study, indicating that elderly patients should not be precluded from lung cancer surgery solely on the basis of their age.

We focused on exploiting detailed radiological parameters and hypothesized that particular radiologic findings would reflect the pulmonary physiologic state even under preserved pulmonary function. As a result, although we excluded clinical interstitial lung disease and abnormal lung function, including chronic obstructive pulmonary disease, ILAs and emphysema were independent factors that predicted PPCs in elderly patients. Previous studies have well documented that abnormal CT findings, such as ILA and emphysema, can present in the elderly without any respiratory symptoms or spirometric abnormalities. While the clinical implications of these findings have not been fully elucidated, recent studies have shown that these abnormal CT findings are associated with rapid lung function decline [28, 29] and increased all-cause mortality [21, 29, 30]. Our study also provided prognostic implications of detecting subclinical radiologic abnormalities, which are associated with the development of PPCs after lung resection surgery. These results implied that radiological abnormalities such as ILA and emphysema could be important determinants that increase the incidence of PPCs in elderly patients even when their lung function is normal. Among ILA and emphysema, ILA remained an independent factor that increased the incidence of PPCs in the present study. Given that emphysematous lung destruction is strongly associated with impaired lung function [31], the predictive role of ILA in PPCs might be stronger than that of emphysema in elderly patients with preserved lung function. Our study suggested that radiologic assessment would assist treatment decision-making even in healthy elderly patients who are candidates for lung resection. We believe that there are real opportunities to find out the possibility of development of PPCs even in this healthy elderly population through preoperative CT. Nevertheless, further study is needed to determine the role of CT biomarkers in predicting PPCs by using longitudinal data.

The extent of lung resection (pneumonectomy or bilobectomy) are also well-known predictive risk factors of PPCs [5]. Pneumonectomy is associated with not only a significant decrease in pulmonary function but also numerous potential complications that involve the pulmonary and cardiovascular systems [32]. Moreover, a previous study reported that the physical quality of life in patients who underwent pneumonectomy was significantly lower than that of the general population [33]. In particular, patients older than 70 years and those with a low preoperative quality of life appear more likely to have an unsatisfactory quality of life at 6 months after pneumonectomy than do other patients [34], and our previous data showed that patients older than 70 years with early stage lung cancer had a worse long-term survival rate after pneumonectomy than younger patients (< 70 years) [35]. Thus, elderly patients scheduled to undergo pneumonectomy would require intensive preoperative cardiopulmonary evaluation as well as an assessment of preoperative physical quality of life.

Among the risk factors we identified, higher ASA classification and lower BMI are well-known risk factors that predict PPC occurrence after lung cancer surgery [36, 37], as a poor physical status, including low muscle mass, are strongly related to ineffective respiration and decreased physical activity after surgery.

The major limitation of our study was its retrospective, single-center design. Thus, patient-centered outcomes, such as the quality of life during the perioperative period, could not be investigated in our study. Further prospective studies are required to assess the impact of the quality of life on PPCs. In addition, as this study was conducted only in patients at a tertiary hospital, the results might not be generalizable to different settings. However, for a single institution study, this is a relatively large cohort of elderly patients.

In conclusion, pulmonary resection for early stage NSCLC seems feasible and should thus be provided to healthy elderly patients after risk factors for PPCs, such as poor performance, lower BMI, ILA on chest CT, and the extent of surgery, are adequately addressed preoperatively. Future prospective studies with long-term follow-up are needed to verify the prognostic significance of these risk factors.

## Additional files

Additional file 1: **Table S1.** Definitions of postoperative pulmonary complications. (DOCX 17 kb)

Additional file 2: **Table S2.** Risk factors associated with PPCs in patients who underwent lobectomy, segmentectomy or wedge resection. (DOCX 15 kb)

## Abbreviations

ARDS: Acute respiratory distress syndrome
ASA: American Society of Anesthesiologists
BMI: Body mass index
CI: Confidence interval
CT: Computed tomography
DLco: Diffusing capacity of the lung for carbon monoxide
FEV_1_: Forced expiratory volume in 1 s
FVC: Forced vital capacity
ILA: Interstitial lung abnormality
NSCLC: Non-small cell lung cancer
OR: Odds ratio
PPC: Postoperative pulmonary complication
