# Supplementary material for: Prevalence of and risk factors for pulmonary complications after curative resection in otherwise healthy elderly patients with early stage lung cancer
Source: Respir Res. 2019 Jul 4;20:136. doi: 10.1186/s12931-019-1087-x (PMC6610954; doi:10.1186/s12931-019-1087-x)
Supplement: Supplementary file 1 — Table S1. Definitions of postoperative pulmonary complications. (DOCX 17 kb) [file 12931_2019_1087_MOESM1_ESM.docx]

Additional file 1: Table S1 Definitions of postoperative pulmonary complications

| Complication | Definition |
| --- | --- |
| Pneumonia | Patient received antibiotics for a suspected respiratory infection  Newly appeared pulmonary infiltration on chest plain radiography  Met at least one of the following criteria:  - New onset or changed sputum  - Fever  - Leukocytosis (>12000/μL)  - Documented pathogen on sputum culture |
| Acute respiratory distress syndrome | Patient met at least one of the following criteria:   - PaO_2_ < 60 mm Hg in room air - PaO_2_ to inspired oxygen fraction < 300 - Arterial oxyhemoglobin saturation measured with pulse oximetry < 90%   Newly appeared pulmonary infiltration on chest plain radiography |
| Respiratory failure | Patient who experienced respiratory failure in the postoperative period requiring mechanical ventilation and/or reintubation |
| Significant atelectasis | Patient who required therapeutic bronchoscopy or reintubation to control atelectasis* |
| Empyema | Pus or infected fluid collected in a pleural space |
| Bronchopleural fistula | Abnormal passage between the bronchus and pleura on chest computed tomography or bronchoscopy |
| Prolonged air leakage | Air leakage lasting for more than 5 days or requiring pleurodesis or chest tube reinsertion |
| Pneumothorax | Air in the pleural cavity which necessitated chest tube reinsertion |

*****Patients with accompanying pneumonia or acute respiratory distress syndrome were not included. PaO_2_: partial pressure of oxygen
