# Supplementary material for: Prevalence of and risk factors for pulmonary complications after curative resection in otherwise healthy elderly patients with early stage lung cancer
Source: Respir Res. 2019 Jul 4;20:136. doi: 10.1186/s12931-019-1087-x (PMC6610954; doi:10.1186/s12931-019-1087-x)
Supplement: Supplementary file 2 — Table S2. Risk factors associated with PPCs in patients who underwent lobectomy, segmentectomy or wedge resection. (DOCX 15 kb) [file 12931_2019_1087_MOESM2_ESM.docx]

Additional file 2: Table S2. Risk factors associated with PPCs in patients who underwent lobectomy, segmentectomy or wedge resection

|  | **Univariate analysis** | | **Multivariate analysis** | |
| --- | --- | --- | --- | --- |
| **Variable** | **OR (95% CI)** | ***p*-value** | **OR (95% CI)** | ***p*-value** |
| Age | 0.96 (0.88–1.06) | 0.412 |  |  |
| Sex, male | 2.10 (1.12–3.94) | **0.021** |  |  |
| Smoking history  (current & former) | 1.86 (1.01–3.45) | **0.046** |  |  |
| ASA classification ≥ 3 | 2.92 (1.02–8.32) | **0.045** |  |  |
| BMI | 0.86 (0.77–0.96) | **0.005** | 0.86 (0.78–0.95) | **0.005** |
| Hemoglobin | 1.11 (0.88–1.39) | 0.362 |  |  |
| Squamous vs. others | 2.18 (1.07–4.46) | **0.032** |  |  |
| ILA | 1.07 (1.02–1.13) | **0.008** | 1.07 (1.02–1.13) | **0.008** |
| Emphysema index | 1.04 (1.01–1.08) | **0.004** |  |  |

ASA: American Society of Anesthesiologists; BMI: body mass index; CI: confidence interval; ILA: interstitial lung abnormality; OR: odds ratio; PPCs: postoperative pulmonary complications
